# Early Glycemic Control With iGlarLixi Versus IDegAsp in Chinese Adults With Type 2 Diabetes: A Post Hoc Analysis of the Soli‐D Study

**DOI:** 10.1111/1753-0407.70171

**Published:** 2025-11-25

**Authors:** Xiaohong Wu, Ying Zhang, Ziling Li, Agustina Alvarez, Felipe Lauand, Lydie Melas‐Melt, Minlu Zhang, Lei Kang, Qin Du, Jie Zhang, Yiming Mu

**Affiliations:** ^1^ Department of Endocrinology Zhejiang Provincial People's Hospital (Affiliated People's Hospital, Hangzhou Medical College) Hangzhou China; ^2^ Department of Endocrinology The Third Affiliated Hospital of Guangzhou Medical University Guangzhou China; ^3^ Department of Endocrinology Inner Mongolia Baogang Hospital Baotou China; ^4^ Sanofi Madrid Spain; ^5^ Sanofi Paris France; ^6^ Ividata Life Sciences Paris France; ^7^ Sanofi Shanghai China; ^8^ Sanofi Beijing China; ^9^ Department of Endocrinology The First Medical Center of PLA General Hospital Beijing China

To the Editor,

1

Early glycemic control provides lifelong benefits in individuals with type 2 diabetes (T2D) [1]; however, < 40% of Chinese adults with T2D achieve glycated hemoglobin (HbA1c) < 7.0% with conventional oral antidiabetic drugs (OADs), rather than early intensive treatment [2, 3]. Following the publication of the phase 3 Soli‐D study [4], the current post hoc analysis evaluated whether iGlarLixi (a fixed‐ratio combination [FRC] comprising insulin glargine 100 U/mL plus lixisenatide) can provide improved early glycemic control at Weeks 8 and 12 compared with the premixed insulin degludec plus insulin aspart (IDegAsp).

In Soli‐D, iGlarLixi demonstrated superior efficacy to IDegAsp among Chinese adults with suboptimally controlled T2D on metformin (± a second OAD) with regard to HbA1c reductions from baseline to Week 24 [4]. In this analysis, changes from baseline in HbA1c, FPG, and body weight, as well as total insulin daily dose were assessed at Weeks 8 and 12, and changes in 7‐point self‐measured plasma glucose (SMPG) and 2‐h PPG, and hypoglycemia event rates were evaluated at Week 12. The proportion of participants who achieved HbA1c < 7.0%, FPG ≤ 7.0 mmol/L, or 2‐h PPG < 10.0 mmol/L were also assessed at Weeks 8 and 12. HbA1c, FPG, and 2‐h PPG data were used to estimate the median time to glycemic target achievement (i.e., the time taken for 50% of participants to reach glycemic target).

Compared with IDegAsp, iGlarLixi was associated with greater reductions from baseline in HbA1c at Week 8 (least squares mean ± standard error difference −0.33 ± 0.06) and Week 12 (−0.32 ± 0.06), and in average 7‐point SMPG (−0.66 ± 0.13) and 2‐h PPG (−1.06 ± 0.16) at Week 12, while changes in FPG remained similar (Table 1). Additionally, iGlarLixi required lower total insulin daily doses and demonstrated body weight benefits over IDegAsp at both timepoints. The hypoglycemia event rate was numerically lower with iGlarLixi versus IDegAsp at Week 12 (Table S1), and iGlarLixi provided higher achievement rates of target HbA1c at Weeks 8 and 12, and target 2‐h PPG at Week 12 compared with IDegAsp (Figure S1). The time to achievement of target HbA1c or target 2‐h PPG was shorter with iGlarLixi than with IDegAsp (Figure S2).

**TABLE 1:** Efficacy outcomes at Weeks 8 and 12 with iGlarLixi and IDegAsp.

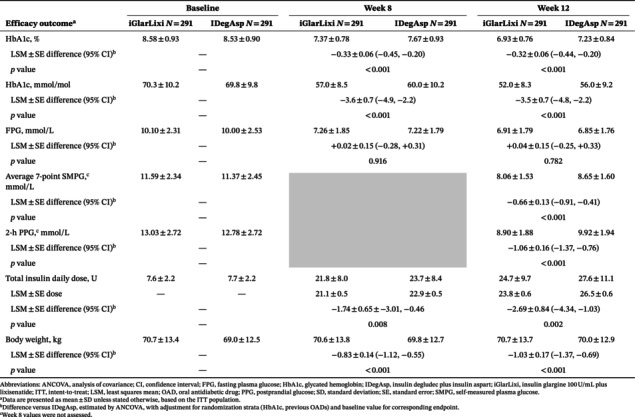

These results at Weeks 8 and 12 are consistent with those previously reported at Week 24 in the primary analysis of Soli‐D [4]. The early glycemic benefits of iGlarLixi over IDegAsp observed in this analysis are most likely attributable to the PPG reductions provided by the lixisenatide component of the FRC. Unlike premixed insulins, the glucagon‐like peptide‐1 receptor agonist (GLP‐1 RA) lixisenatide acts by delaying gastric emptying and reducing glucagon secretion, providing greater reductions in PPG excursions. Based on post‐trial monitoring data from the UK Prospective Diabetes Study in adults with newly diagnosed T2D [1], early and effective glycemic control with iGlarLixi may reduce the lifetime risk of diabetes‐related complications and mortality.

In conclusion, once‐daily iGlarLixi therapy provides improved early glycemic control (after 8 or 12 weeks) compared with IDegAsp in Chinese adults with suboptimally controlled T2D. Therefore, iGlarLixi may be a valuable treatment option in this setting, providing comprehensive glycemic control and body weight benefits, with a low risk of hypoglycemia.

## Author Contributions

All named authors meet the International Committee of Medical Journal Editors (ICMJE) criteria for authorship for this article and had full access to all the data in this study, and take full responsibility for the integrity of the data and accuracy of the data analysis. Concept and design: Agustina Alvarez, Yiming Mu, Felipe Lauand, Qin Du, Lei Kang. Conduct and data collection: Agustina Alvarez, Yiming Mu, Xiaohong Wu, Ying Zhang, Ziling Li. Data analysis: Agustina Alvarez, Minlu Zhang, Lydie Melas‐Melt. Writing – original draft: Jie Zhang. All authors had final responsibility for approving the published version.

## Funding

The Soli‐D study was funded by Sanofi.

## Ethics Statement

The study was conducted in accordance with the Declaration of Helsinki and the International Council for Harmonization of Technical Requirements for Pharmaceuticals for Human Use guidelines for Good Clinical Practice.

## Consent

All participants provided written informed consent prior to the start of the study.

## Conflicts of Interest

X.W., Y.Z. and M.Z. have nothing to disclose. A.A., F.L., L.K., Q.D., and J.Z. are Sanofi employees. L.M.‐M. is an employee of Ividata Life Sciences, contracted by Sanofi. Y.M. reports having received honoraria and personal fees from Eli Lilly Diabetes, Novo Nordisk, and Sanofi outside the submitted work.

## Supporting information


**TABLE S1:** Hypoglycemia outcomes at Week 12.
**FIGURE S1:** The proportion of participants who achieved HbA1c < 7.0%, FPG ≤ 7.0 mmol/L, and 2‐h PPG < 10.0 mmol/L with iGlarLixi versus IDegAsp at early (Week 8 and/or Week 12) study visits. CI, confidence interval; FPG, fasting plasma glucose; HbA1c, glycated hemoglobin; IDegAsp, insulin degludec plus insulin aspart; iGlarLixi, insulin glargine 100 U/mL plus lixisenatide; OR, odds ratio; PPG, postprandial glucose. aEstimated by logistic regression, adjusted for randomization strata and corresponding baseline value. bParticipants without glycemic endpoint data at corresponding visits were considered non‐responders.
**FIGURE S2:** Kaplan–Meier curves of median time to first target (a) HbA1c, (b) FPG, and (c) 2‐h PPG with iGlarLixi versus IDegAsp. CI, confidence interval; FPG, fasting plasma glucose; HbA1c, glycated hemoglobin; HR, hazard ratio; IDegAsp, insulin degludec plus insulin aspart; iGlarLixi, insulin glargine 100 U/mL plus lixisenatide; OADs, oral antidiabetic drugs; PPG, postprandial glucose. aEstimated by stratified Cox regression model, stratified by randomization strata (HbA1c, previous OADs) and using treatment arm as the model factor.

## Data Availability

Qualified researchers may request access to participant‐level data and related documents. Participant‐level data will be anonymized, and study documents will be redacted to protect the privacy of trial participants. Further details on Sanofi's data sharing criteria, eligible studies, and process for requesting access can be found at https://www.vivli.org.
