# Supplementary material for: Early Glycemic Control With iGlarLixi Versus IDegAsp in Chinese Adults With Type 2 Diabetes: A Post Hoc Analysis of the Soli‐D Study
Source: J Diabetes. 2025 Nov 25;17(11):e70171. doi: 10.1111/1753-0407.70171 (PMC12647919; doi:10.1111/1753-0407.70171)

**Early Glycemic Control with iGlarLixi versus IDegAsp in Chinese Adults with Type 2 Diabetes: A Post Hoc Analysis of the Soli-D Study**

**Authors:** Xiaohong WU^1^, Ying ZHANG^2^, Ziling LI^3^, Agustina ALVAREZ^4^, Felipe LAUAND^5^, Lydie MELAS-MELT^6^, Minlu ZHANG^7^, Lei KANG^8^, Qin DU^7^, Jie ZHANG^8^, Yiming MU^9^

**Affiliations:** ^1^Department of Endocrinology, Zhejiang Provincial People’s Hospital (Affiliated People’s Hospital, Hangzhou Medical College), Hangzhou, China; ^2^Department of Endocrinology, The Third Affiliated Hospital of Guangzhou Medical University, Guangzhou, China; ^3^Department of Endocrinology, Inner Mongolia Baogang Hospital, Baotou, China; ^4^Sanofi, Madrid, Spain; ^5^Sanofi, Paris, France; ^6^Ividata Life Sciences, Paris, France; ^7^Sanofi, Shanghai, China; ^8^Sanofi, Beijing, China; ^9^Department of Endocrinology, The First Medical Center of PLA General Hospital, Beijing, China

**Correspondence:** Yiming Mu, Department of Endocrinology, The First Medical Center of PLA General Hospital, 28 Fuxing Road, Haidian District, Beijing 100853, China. Tel: 010-66887329; Fax: 010-68182255; Email: muyiming@301hospital.com.cn

# SUPPORTING INFORMATION

## TABLE S1.

Hypoglycemia outcomes at Week 12.

|  | **iGlarLixi**  **(N=290)** | **IDegAsp**  **(N=291)** |
| --- | --- | --- |
| **Hypoglycemia incidence** |  |  |
| Any hypoglycemia, n (%) | 56 (19.3) | 59 (20.3) |
| OR (95% CI)† | 0.87 (0.53, 1.41) | |
| *p*-value | 0.573 | |
| ADA Level 1 hypoglycemia,‡ n (%) | 51 (17.6) | 55 (18.9) |
| OR (95% CI)† | 0.89 (0.54, 1.47) | |
| *p*-value | 0.641 | |
| ADA Level 2 hypoglycemia,§ n (%) | 7 (2.4) | 13 (4.5) |
| OR (95% CI)† | 0.40 (0.12, 1.30) | |
| *p*-value | 0.126 | |
| ADA Level 3 hypoglycemia,¶ n (%) | 0 | 0 |
| **Hypoglycemia event rate** |  |  |
| Total participant years | 65.34 | 67.17 |
| No. of any hypoglycemia events (rate per PY) | 92 (1.41) | 142 (2.11) |
| RR (95% CI)# | 0.67 (0.43, 1.04) | |
| *p*-value | 0.076 | |
| No. of ADA Level 1 events‡ (rate per PY) | 85 (1.30) | 123 (1.83) |
| RR (95% CI)# | 0.71 (0.45, 1.12) | |
| *p*-value | 0.138 | |
| No. of ADA Level 2 events§ (rate per PY) | 7 (0.11) | 19 (0.28) |
| RR (95% CI)# | 0.39 (0.14, 1.07) | |
| *p*-value | 0.069 | |
| No. of ADA Level 3 events¶ (rate per PY) | 0 | 0 |

Abbreviations: ADA, American Diabetes Association; CI, confidence interval; HbA1c, glycated hemoglobin; IDegAsp, insulin degludec plus insulin aspart; iGlarLixi, insulin glargine 100 U/mL plus lixisenatide; No., number; OADs, oral antidiabetic drugs; OR, odds ratio; PY, participant-year; RR, rate ratio.
†Determined by logistic regression, adjusted for treatment group and randomization strata (HbA1c, previous OADs).
‡Defined as a measurable plasma glucose ≥3.0 mmol/L to <3.9 mmol/L.
§Defined as a measurable plasma glucose <3.0 mmol/L.
¶Defined as a severe hypoglycemia event characterized by altered mental and/or physical functioning requiring assistance.
#Determined by negative binomial regression, adjusted for treatment group and randomization strata (HbA1c, previous OADs), with the logarithm of the duration (in years) of the open-label, randomized treatment period used as an offset variable.

## FIGURE S1

The proportion of participants who achieved HbA1c <7.0%, FPG ≤7.0 mmol/L, and 2-hour PPG <10.0 mmol/L with iGlarLixi versus IDegAsp at early (Week 8 and/or Week 12) study visits. CI, confidence interval; FPG, fasting plasma glucose; HbA1c, glycated hemoglobin; IDegAsp, insulin degludec plus insulin aspart; iGlarLixi, insulin glargine 100 U/mL plus lixisenatide; OR, odds ratio; PPG, postprandial glucose. ^a^Estimated by logistic regression, adjusted for randomization strata and corresponding baseline value. ^b^Participants without glycemic endpoint data at corresponding visits were considered non-responders.


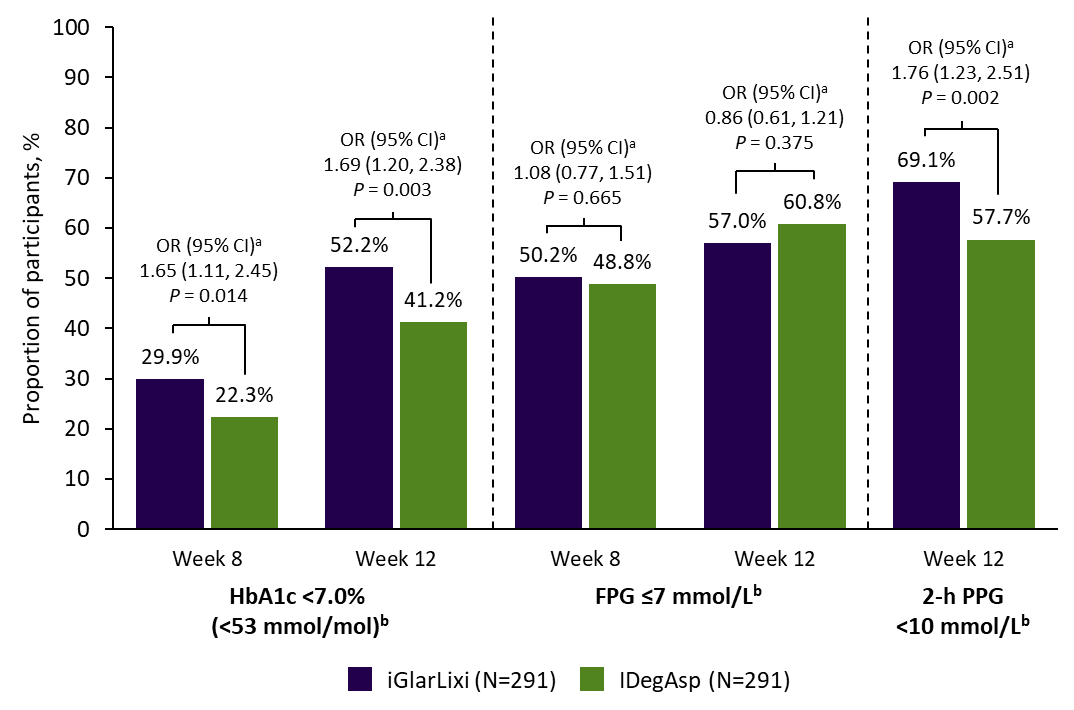


## FIGURE S2

Kaplan-Meier curves of median time to first target (a) HbA1c, (b) FPG, and (c) 2-hour PPG with iGlarLixi versus IDegAsp. CI, confidence interval; FPG, fasting plasma glucose; HbA1c, glycated hemoglobin; HR, hazard ratio; IDegAsp, insulin degludec plus insulin aspart; iGlarLixi, insulin glargine 100 U/mL plus lixisenatide; OADs, oral antidiabetic drugs; PPG, postprandial glucose. ^a^Estimated by stratified Cox regression model, stratified by randomization strata (HbA1c, previous OADs) and using treatment arm as the model factor.


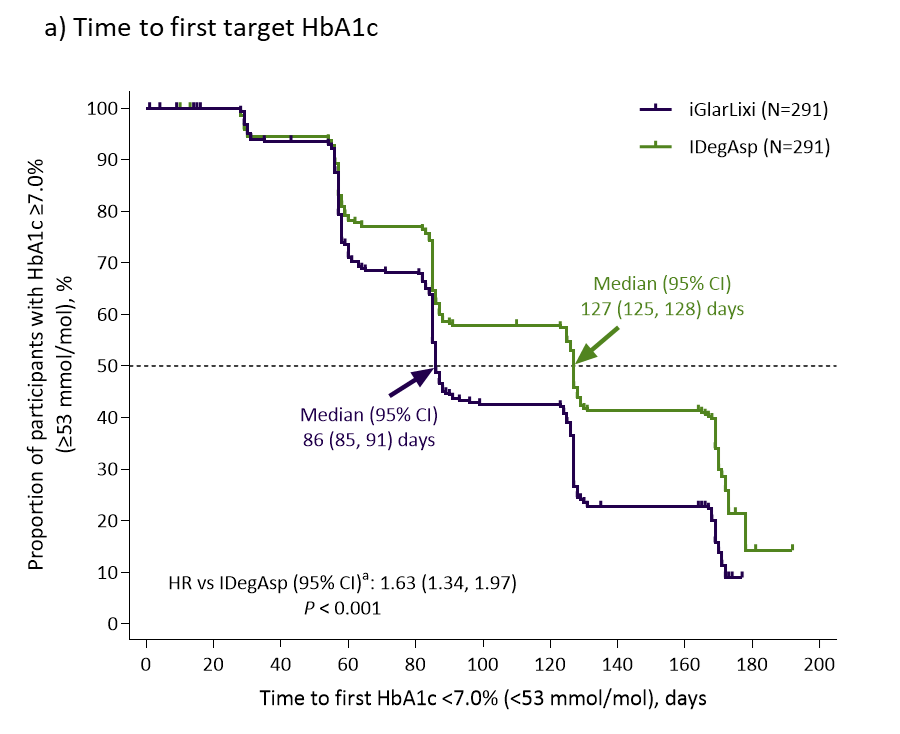


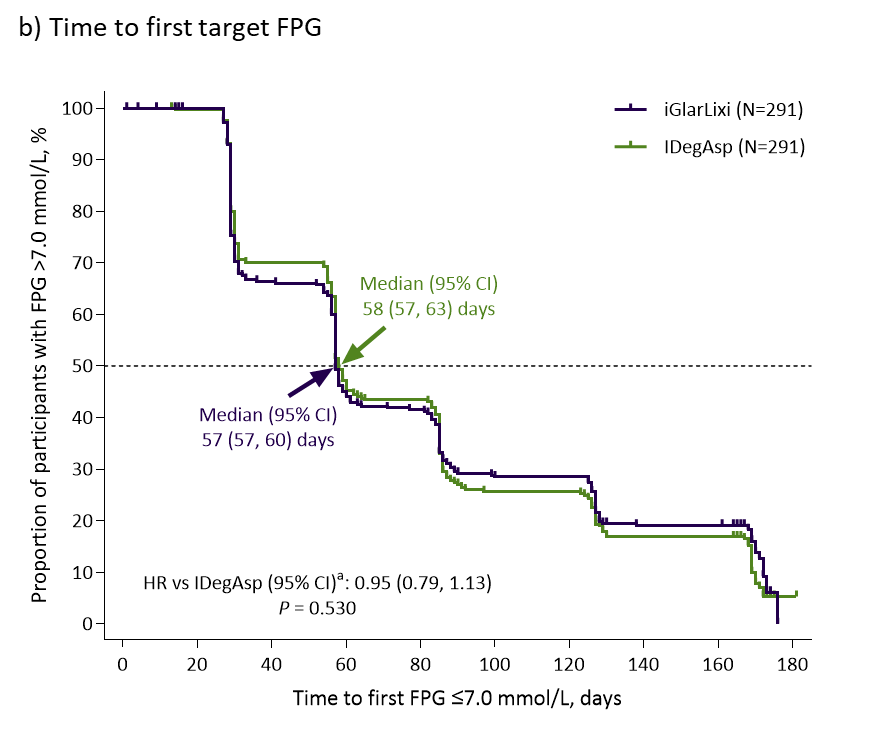


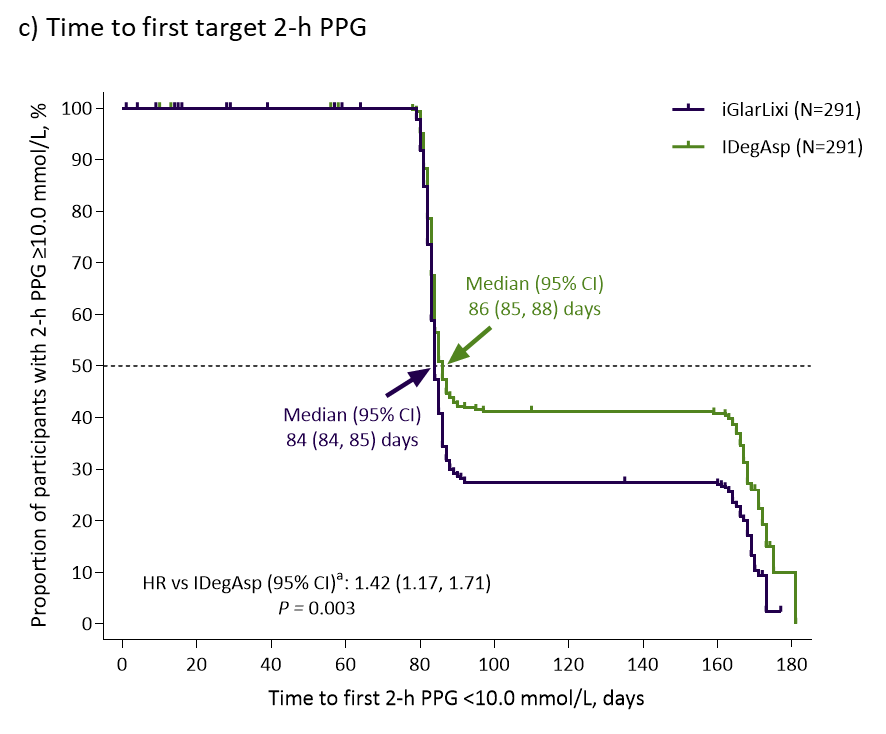

Supplement: Supplementary file 1 — TABLE S1: Hypoglycemia outcomes at Week 12. FIGURE S1: The proportion of participants who achieved HbA1c < 7.0%, FPG ≤ 7.0 mmol/L, and 2‐h PPG < 10.0 mmol/L with iGlarLixi versus IDegAsp at early (Week 8 and/or Week 12) study visits. CI, confidence interval; FPG, fasting plasma glucose; HbA1c, glycated hemoglobin; IDegAsp, insulin degludec plus insulin aspart; iGlarLixi, insulin glargine 100 U/mL plus lixisenatide; OR, odds ratio; PPG, postprandial glucose. aEstimated by logistic regression, adjusted for randomization strata and corresponding baseline value. bParticipants without glycemic endpoint data at corresponding visits were considered non‐responders. FIGURE S2: Kaplan–Meier curves of median time to first target (a) HbA1c, (b) FPG, and (c) 2‐h PPG with iGlarLixi versus IDegAsp. CI, confidence interval; FPG, fasting plasma glucose; HbA1c, glycated hemoglobin; HR, hazard ratio; IDegAsp, insulin degludec plus insulin aspart; iGlarLixi, insulin glargine 100 U/mL plus lixisenatide; OADs, oral antidiabetic drugs; PPG, postprandial glucose. aEstimated by stratified Cox regression model, stratified by randomization strata (HbA1c, previous OADs) and using treatment arm as the model factor. [file JDB-17-e70171-s001.docx]
